# Collagen scaffolds functionalized with triple-helical peptides support 3D HUVEC culture

**DOI:** 10.1093/rb/rbaa025

**Published:** 2020-08-18

**Authors:** Jean-Daniel Malcor, Emma J Hunter, Natalia Davidenko, Daniel V Bax, Ruth Cameron, Serena Best, Sanjay Sinha, Richard W Farndale

**Affiliations:** r1 Department of Biochemistry, University of Cambridge, Cambridge CB2 1QW, UK; r2 Department of Materials Science and Metallurgy, University of Cambridge, Cambridge CB3 0FS, UK; r3 Division of Medicine and Wellcome Trust, Medical Research Council Cambridge Stem Cell Institute, University of Cambridge, Cambridge CB2 0AW, UK

**Keywords:** collagen biomaterial, endothelial cells, 3D cell scaffold, collagen-mimetic peptides

## Abstract

Porous biomaterials which provide a structural and biological support for cells have immense potential in tissue engineering and cell-based therapies for tissue repair. Collagen biomaterials that can host endothelial cells represent promising tools for the vascularization of engineered tissues. Three-dimensional collagen scaffolds possessing controlled architecture and mechanical stiffness are obtained through freeze–drying of collagen suspensions, followed by chemical cross-linking which maintains their stability. However, cross-linking scaffolds renders their biological activity suboptimal for many cell types, including human umbilical vein endothelial cells (HUVECs), by inhibiting cell–collagen interactions. Here, we have improved crucial HUVEC interactions with such cross-linked collagen biomaterials by covalently coupling combinations of triple-helical peptides (THPs). These are ligands for collagen-binding cell-surface receptors (integrins or discoidin domain receptors) or secreted proteins (SPARC and von Willebrand factor). THPs enhanced HUVEC adhesion, spreading and proliferation on 2D collagen films. THPs grafted to 3D-cross-linked collagen scaffolds promoted cell survival over seven days. This study demonstrates that THP-functionalized collagen scaffolds are promising candidates for hosting endothelial cells with potential for the production of vascularized engineered tissues in regenerative medicine applications.

## Introduction

Porous biomaterials that provide cells with a 3D structure and guide tissue formation are often used in tissue engineering [1]. Decellularized matrix constitutes an attractive cellular scaffold that preserves the natural complexity of the extracellular matrix (ECM) [2, 3]. However, its structure is not necessarily suitable for efficient cell re-seeding, and issues relating to organ availability and pathogen transfer may arise. Recently, proteins isolated from the ECM have shown great promise in reproducing its mechanical properties at reduced cost. As the main constituent of the ECM, collagen naturally provides both biological cues and a structural support for cells through a dense fibrillar network [4, 5]. Porous collagen scaffolds, frequently obtained through freeze–drying of a collagen suspension [6, 7], are thus widely used to manufacture engineered tissues [8], with applications in regenerative medicine [9] or *in vitro* modelling of tissues [10]. However, collagen-based materials dissolve over time and contract in cell culture conditions [11]. To achieve adequate mechanical properties, collagen scaffolds are frequently chemically cross-linked using carbodiimide reagents, often 1-ethyl-3-(3-dimethylamino-propyl)carbodiimide (EDC) and *N*-hydroxysuccinimide (NHS) [12]. EDC/NHS cross-linking allows control over stiffness and elasticity, making it possible to match the physical characteristics of the targeted healthy tissue. In our laboratory, we routinely EDC/NHS cross-link scaffolds with a 5/2/1 molar ratio of EDC/NHS/collagen, referred to as 100% cross-linking in our previous studies [13]. This cross-linking method raised the tensile Young’s modulus of 2D collagen films from 5.7 to 31 MPa [14] and the compressive modulus of 3D scaffold from 1.2 to 6.2 kPa [15], leading to stable collagen biomaterials.

EDC/NHS forms amide bonds between collagen molecules by bridging aspartate or glutamate with lysine residues, some of which are involved in the binding of cell-surface receptors and cell-secreted proteins to collagen. These include collagen-binding integrins, which are essential for initiating cellular attachment; discoidin domain receptor (DDR) 2, which plays an important role in wound repair [16, 17] and von Willebrand Factor (VWF), a key component of thrombus formation [18]. As a result, the chemical modification of the collagen sequence associated with EDC/NHS cross-linking results in diminished cell–scaffold interactions, leading to poor attachment of C2C12, HT1080 and Rugli experimental cell models [14, 19, 20]. To overcome this limiting issue, RGD peptides, antibodies or growth factors have been incorporated into collagen biomaterials to stimulate cell responses via other pathways [21–23]. Here, we have instead specifically replicated interactions between cells and native collagen by loading scaffolds with triple-helical peptides (THPs). THPs are composed of three peptide strands that adopt the triple-helical conformation required for collagen-binding protein recognition and which mimics the native structure of collagen. THPs containing collagen-derived sequences such as GFOGER (targeting collagen-binding integrins) or GPRGQOGVNleGFO [where Nle is Norleucine, targeting DDR 1 and 2, VWF and Secreted Protein, Acidic, Rich in Cysteine (SPARC)] have enhanced cell function on 2D collagen films [19, 20]. Here, we demonstrate that this technology can be applied to 3D constructs.

Poor vascularization of engineered tissues represents a major obstacle to their clinical use [24, 25]. Vascularization requires the formation of a quiescent non-thrombogenic surface constituted of endothelial cell monolayers [26]. The aim of this study is to improve endothelial cell support, a prominent goal for cell-based therapies. Endothelial cells are complex cells that are highly sensitive to cell culture conditions and interact with various ECM macromolecules *in vivo*, including collagen (mainly type IV), proteoglycans, glycosaminoglycans, laminin and fibronectin [27]. Robust attachment to the ECM is notably required to prevent ablation under shear stress and subsequent exposure of the underlying matrix to circulating platelets and consequent thrombus formation. The main challenge of this work was to fabricate scaffolds with high affinity for endothelial cells without the need to alter cell culture conditions, which may trigger unwanted activation or changes in phenotype. For this purpose, collagen substrates functionalized with THPs that are ligands for collagen-binding proteins were seeded with human umbilical vein endothelial cells (HUVECs), chosen as models for endothelial cells. While EDC/NHS cross-linking was required to ensure the structural integrity of collagen biomaterials and HUVEC short-term survival, THPs were essential to maintain HUVEC culture. Specifically, THPs significantly improved HUVEC affinity for the collagen substrate, proliferation and survival, enabling HUVECs to populate scaffolds. First, our results provide valuable insight into the importance of cell–collagen interactions on endothelial cell responses. Second, we have demonstrated that THP-functionalized EDC/NHS cross-linked collagen scaffolds are biologically active matrices which can support HUVEC function over extended culture durations. Such scaffolds constitute promising biomaterials for the generation of engineered tissues and have great potential for translation to tissue repair research.

## Materials and methods

### THP synthesis

9-Fluorenylmethoxycarbonyl (Fmoc) protected amino acids were supplied by AGTC Bioproducts. Fmoc-protected 6-Aminohexanoic acid (Ahx) was supplied by Merck. All other reagents were purchased from Sigma-Aldrich. (GPP)_5_GFOGER(GPP)_5_, (GPP)_5_GLOGEN(GPP)_5_, Ahx-(GPP)_5_GMOGER(GPP)_5_ and Ahx-(GPP)_5_GPRGQOGVNleGFO(GPP)_5_ were synthesized and end-stapled as previously described [19, 20], yielding diazirine-coupled end-stapled *GFOGER, GLOGEN, GMOGER* and *VWFIII_Nle_*, respectively. *GFOGER* (0.1 × 10^−6 ^mol), 2-tert-Butyl-1,1,3,3-tetramethylguanidine (3 × 10^−6 ^mol) and 5(6)-carboxyfluorescein (FITC) succinimidyl ester (1.2 × 10^−6 ^mol) were dissolved in 200 µl of dimethylformamide and left overnight in the dark at 40°C. Then, 2 ml of water was added and the mixture was freeze–dried. The crude product was dissolved in 0.5 ml water, dialyzed and freeze–dried to yield the *GFOGER–FITC* fluorescent compound.

### HUVEC culture conditions

Pooled HUVECs (Promocell, Heidelberg, Germany) were cultured in Endothelial Cell Growth Medium 2 (EGM-2, Promocell) at 37°C with 5% CO_2_. HUVECs were used between passages 3 and 5. The 70–90% confluent HUVECs were washed with PBS and detached with tryplE for 5 min at room temperature. TryplE was quenched with 1 ml of PBS, and cells were spun down at 280 g for 4 min and re-suspended in EGM-2. 

### Preparation of collagen films and scaffolds

THP-functionalized collagen films [14, 19] and collagen scaffolds [28] were prepared and EDC/NHS cross-linked as previously described (referred to as 100% cross-linking in our previous work). The 2 mm thick and 6 mm wide cylinder-shaped cross-linked scaffolds, weighing approximately 1 mg, were cut using a disposable biopsy punch and a vibrating microtome tissue slicer. Scaffolds were incubated with peptides diluted to 10 μg/ml in 0.01 M AcOH (for concentration studies, FITC-fluorescent peptides were added at concentrations between 0 and 500 μg/ml), gently compressed until all air bubbles were removed and left in solution for 30 min in the dark. Scaffolds were placed under a long-wavelength UV lamp (Blak-Ray B100AP, 365 nm wavelength) for 5 min, turned upside down and exposed to UV for a further 5 min. Scaffolds were washed by gently compressing with citrate buffer (pH 3) 3 × 2 min and PBS 3 × 2 min. Scaffold architecture was visualized by Scanning Electron Microscopy (SEM, JEOL 5800). Pore size, strut thickness and porosity were analysed by X-ray microtomography (Skyscan 1072 Micro-CT), with a 28 kV/164 μA X-ray source. Cross-sections were generated using a full cone beam Feldkamp reconstruction algorithm. Following functionalization with *GFOGER–FITC*, FITC fluorescence intensity in scaffold cross-sections was quantified in five randomly selected fields of view within each of four repeats using a Leica DM6000 microscope. Prior to seeding with HUVECs, scaffolds were sterilized by exposure to UV for 30 min.

### HUVEC response on collagen films

500 µl of cell solution at 50 000 cells/ml were added to collagen films prepared in 12-well plates and incubated at 37°C with 5% CO_2_. Spreading assays were performed as described previously [19]. The mean surface covered by cells was quantified on 10 randomly selected fields of view within each of three repeats (or five fields of view in the presence of EDTA) acquired on an Olympus FV300 laser-scanning confocal microscope. Proliferation assays were performed 22 h after seeding using a Click-iT™ EdU Alexa-Fluor 488 kit (Thermo Scientific). 20 µl per well of 5-ethynyl-2’-deoxyuridine (EdU) at 1 mM were added and cells were incubated at 37°C with 5% CO_2_ for further 2 h in the dark. Cells were fixed with 3% paraformaldehyde for 15 min, washed with 3 × 500 µl of 3% BSA in PBS, permeabilized with 1 ml of 0.5% Triton X-100 in PBS for 5 min and washed with 2 × 500 µl of 3% BSA in PBS. 500 µl per well of Click-iT solution together with 1:2000 of Hoechst 33342 were added for 30 min in the dark. Cells were washed with 3 × PBS and kept at 4°C in the dark before imaging. Ten randomly selected fields of view within each of three repeats were acquired on a Leica DM6000 FS or a Zeiss Axio Z1 microscope.

### Lactate dehydrogenase and vascular cell adhesion protein 1 release

300 μl of HUVECs at 100 000 cells/ml in EGM-2 were deposited on top of scaffolds and left at 37°C with 5% CO_2_ for 24 h. For cell death assays, 100 μl of cell culture media was sampled and transferred into 96-well plates containing 50 μl of lactate dehydrogenase (LDH) Cytotoxicity Detection KitPlus (Roche). Absorbance was measured at 490 nm after 30 min. Controls included HUVECs cultured in EGM-2 on empty tissue culture plates coated with BSA or lysed in 0.5% Triton overnight. For vascular cell adhesion protein 1 (VCAM-1) release in media quantitation, 96-well plates were coated with 100 μl of anti-VCAM-1 capture antibody at 2 μg/ml (R&D Systems) overnight. 100 μl of cell culture media from HUVECs were added to wells and left for 1 h. Wells were washed with 3 × 1% BSA in PBS, 100 μl of anti-VCAM-1 detection antibody conjugated to Biotin (R&D Systems) at 200 ng/ml was added for 1 h, wells were washed with 3 × 1% BSA in PBS, 100 μl of Steptavidin-HRP was added for 20 min in the dark and wells were washed with 4 × 1% BSA in PBS. 100 μl of TMB substrate (Thermo Scientific) were added, the reaction was quenched with 100 μl of 2.5 M sulphuric acid and the absorbance was read at 450 nm. Corresponding VCAM concentrations were calculated using a dose curve generated with VCAM (R&D Systems) at concentrations from 0 to 1000 pg/ml. Controls included HUVECs cultured in EGM-2 on empty tissue culture plates coated with BSA overnight without supplements or with 20 ng/ml of TNF-α.

### Cell distribution in collagen scaffolds

200 μl of HUVECs at 500 000 cells/ml in EGM-2 were deposited on top of scaffolds and left at 37°C with 5% CO_2_. Media was replaced with fresh EGM-2 after 24 h and every two days after that. At day 1, 4 or 7, cells were fixed with 3% paraformaldehyde for 15 min and scaffolds were washed with 4 × 1% BSA in PBS while gently compressing the scaffolds. 200 μl of Hoechst 33342 1:2000 and Rhodamine–Phalloidin 1:1000 in PBS were added and left for 30 min in the dark. Scaffolds were washed with 3 × 1% BSA in PBS and were kept at 4°C in the dark before imaging. Vertical sequences (Z-stacks) were acquired on a Zeiss LSM700 upright confocal microscope over a total depth of 50 μm. Eight fields of view from the top of scaffolds for each condition within each of three repeats and six tiled images of 640 μm vertically and 2945 μm horizontally from cross-sections of scaffolds within each of four repeats were acquired. For median cell depth calculations, the distance between the top and the bottom of the scaffold was normalized to 2 mm in each tile image. The median distance of each individual cell from the top of the scaffold was calculated.

### Platelet endothelial cell adhesion molecule 1 and VWF staining

HUVECs were seeded on scaffolds, cultured for 7 days, fixed and stained with Hoechst 33342 and Rhodamine–Phalloidin as described above. Scaffolds were immersed in 300 μl of 0.5% Triton in water for 5 min, washed with 3 × 1% BSA in PBS, blocked with 3% filtered BSA for 1 h and washed with 3 × 0.1% Tween-20 in PBS. Rabbit anti-VWF (Abcam, UK) and goat anti-platelet endothelial cell adhesion molecule 1 (anti-PECAM) (Santa Cruz, USA) were added to scaffolds at 1:200 and 1:1000 dilutions, respectively, for 1 h 30 min in PBS with 0.1% BSA. Scaffolds were washed with 3 × 0.1% Tween-20 in PBS. Donkey anti-rabbit coupled to Alexa Fluor 488 (Jackson ImmunoResearch, UK) and donkey anti-goat coupled to Alexa Fluor 680 (Jackson ImmunoResearch, UK) were added to scaffolds at 1:500 dilution for 1 h in PBS with 0.1% BSA. Scaffolds were washed with 4 × 1% BSA in PBS and kept at 4°C in the dark before imaging. Images were acquired on a Zeiss LSM700 upright confocal microscope over a total depth of 105 μm (30 slices with a 3.5 μm interval).

### Statistical analysis

Microscope images for quantitative analysis were exported and analysed using ImageJ 1.51 (National Institutes of Health). Values shown are mean ± standard error of the mean from up to four independent biological repeats. Mean values were compared using one-way ANOVA with Student’s *t*-tests using Prism software (GraphPad, San Diego). For grouped analysis in Fig. 5B, two-way ANOVA was performed with Bonferroni post-test. All conditions were compared with cross-linked films or scaffolds without peptides. **** on figure denotes *P* < 0.0001, *** denotes *P* < 0.001, ** denotes *P* < 0.01, * denotes *P* < 0.05 and ns denotes non-significant.

## Results

### HUVEC interactions with THP-functionalized 2D collagen films

**Figure 1:**
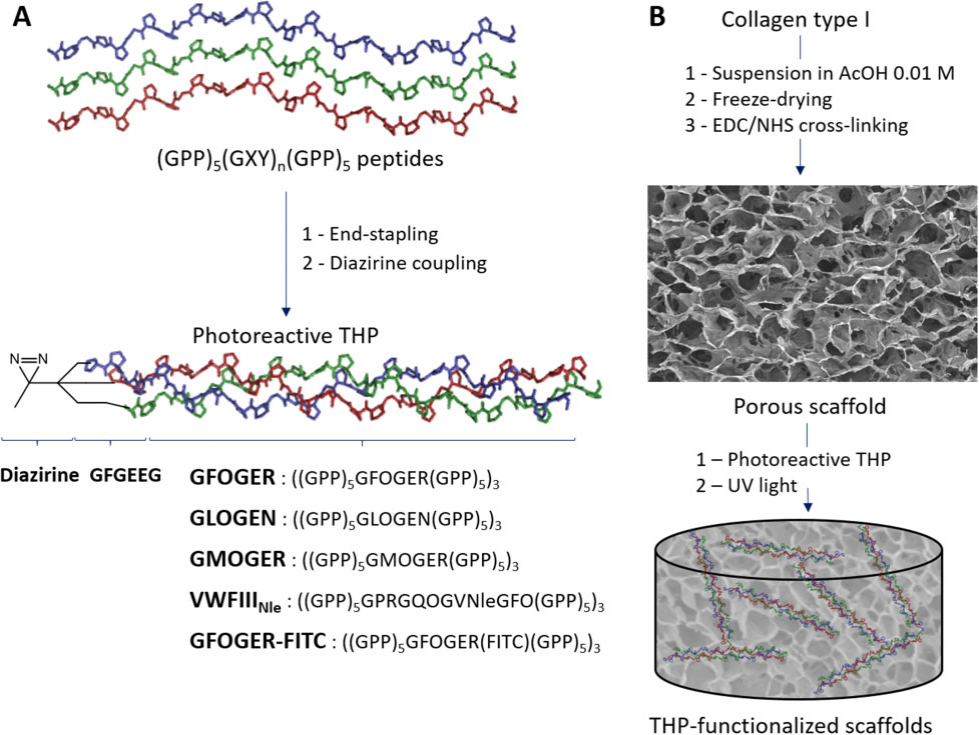
Scaffold functionalization with THPs (**A**) THP synthesis pathway. Peptides containing active sequences GFOGER (targeting preferentially α2β1 and α11β1 integrins), GLOGEN (targeting preferentially α1β1 and α10β1 integrins), GPRGQOGVNleGFO (targeting DDR1, DDR2, VWF, SPARC) or GMOGER (targeting all collagen-binding integrins with moderate affinity) flanked by 5 GPP triplets were synthesized, end-stapled and coupled to diazirine to yield photoreactive THPs *GFOGER*, *GLOGEN*, *VWFIII_Nle_* and *GMOGER*, respectively. (**B**) Scheme of collagen scaffold fabrication and functionalization with THPs. A collagen type I suspension is freeze–dried and cross-linked with EDC/NHS (representative SEM micrograph is shown). THPs were incubated on cross-linked scaffolds and covalently linked to the collagen backbone upon exposure to long-wavelength UV light to obtain THP-functionalized scaffolds.

To promote HUVEC interaction with EDC/NHS cross-linked collagen films or scaffolds, we functionalized these materials with THPs containing active recognition motifs for collagen-binding proteins. These fall into two categories: *GFOGER*, *GLOGEN* and *GMOGER* recognizing the collagen-binding integrins α1β1, α2β1, α10β1 and α11β1; and *VWFIII_Nle_* recognizing DDR1, DDR2, SPARC and VWF. As described previously [19], THPs were end-stapled and a diazirine photoreactive group was grafted to enable covalent linkage to cross-linked films upon UV treatment (Fig. 1). Each photoreactive peptide was introduced at a concentration of 2.5 μg/ml. When *VWFIII_Nle_* was combined with *GFOGER* or *GLOGEN*, this amounts to a total peptide concentration of 5 μg/ml. When a single active peptide is present, we added 2.5 μg/ml of GPP10, a biologically inert THP, to consistently maintain a total peptide concentration of 5 μg/ml. THPs were then covalently linked to collagen films by exposure to long-wavelength UV light to secure THP attachment and prevent elution.

**Figure 2:**
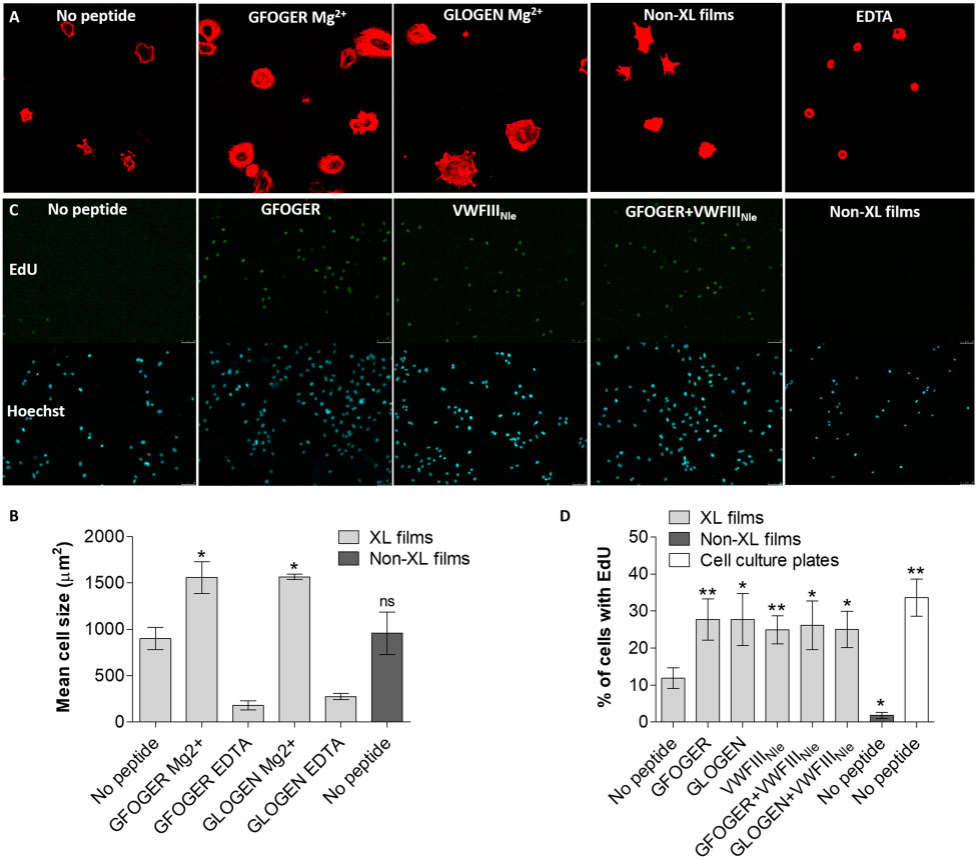
HUVECs seeded on collagen films non-cross-linked (non-XL) or EDC/NHS cross-linked (XL), without peptide or with *GFOGER*, *GLOGEN*, *VWFIII_Nle_*, *GFOGER* + *VWFIII_Nle_* or *GLOGEN* + *VWFIII_Nle_* (**A**) HUVEC spreading in the presence of magnesium or EDTA. Cells were fixed and stained with Rhodamine–Phalloidin. Representative fields of view are shown. HUVECs seeded on films with *GFOGER* or *GLOGEN* with magnesium displayed actin polymerization and filopodia/lamellipodia extensions. (**B**) Mean cell area. Significance for each condition compared with cross-linked films without peptide is shown. *GFOGER* and *GLOGEN* significantly increased the mean cell area in a magnesium-dependent manner. (**C**) HUVEC uptake of EdU after 24 h. Cells were fixed and stained with Hoechst 33342 and EdU-Alexa Fluor-488. Representative fields of view are shown. (**D**) Percentage of EdU-positive cells 24 h after seeding. Significance for each condition compared with cross-linked films without peptide is shown. HUVECs did not proliferate on non-cross-linked collagen films and EDC/NHS cross-linking resulted in a rise of the proliferation rate. Cell growth was further enhanced by THPs.

HUVECs were initially seeded on non-cross-linked and EDC/NHS cross-linked collagen films, a convenient platform to model the inner pore surface present in 3D scaffolds. Although cross-linking was essential to prevent film dissolution in aqueous solution over time, cell spreading (visualized by actin staining, Fig. 2A) was not significantly different without and with EDC/NHS treatment (959 ± 230 and 899 ± 120 µm^2^ mean cell area, respectively, Fig. 2B). Cell attachment and spreading to native collagen primarily occurs through collagen-binding integrins in a magnesium-dependent manner. We attributed weak cell spreading on cross-linked films to the loss of integrin recognition, that is largely ablated by EDC/NHS treatment but restored by coating with *GFOGER*, as shown in our previous study [19]. HUVECs were then seeded in the presence of Mg^2+^ or EDTA (a magnesium chelator) on cross-linked collagen films functionalized with *GLOGEN* and *GFOGER*. Films coated with *GFOGER* or *GLOGEN* supported strong actin polymerization accompanied by filopodia and lamellipodia extensions in the presence of magnesium. THPs induced a significant increase in cell size (one-way ANOVA, *P* < 0.001), both with *GFOGER* (1561 ± 172 µm^2^, *P* < 0.05) and *GLOGEN* (1568 ± 29 µm^2^, *P* < 0.05). With EDTA, very few cells were adherent and were rounded when present (236 ± 21 µm^2^ cell surface for *GFOGER*, 274 ± 32 µm^2^ for *GLOGEN*), demonstrating that cell spreading occurs in a cation-dependent manner presumably through collagen-binding integrins.

Next, HUVEC proliferation 24 h after seeding on collagen films was investigated. EdU internalized in DNA of cells undergoing division was detected by coupling to Alexa Fluor 488 and all cell nuclei were stained with Hoechst 33342 (Fig. 2C). The percentage of EdU positive cells was calculated (one-way ANOVA, *P* < 0.001, Fig. 2D) with HUVECs seeded on tissue culture plastic as positive controls (33.65 ± 5.00%). HUVEC were more proliferative on cross-linked films (11.87 ± 2.83%) than on untreated films (1.80 ± 0.83%, *P* < 0.05), highlighting the importance of collagen biomaterial cross-linking as a pre-requisite for cellular culture. To further improve HUVEC proliferation, films were functionalized with *GFOGER*, *GLOGEN* or *VWFIII_Nle_*, as well as combinations of *VWFIII_Nle_* with *GFOGER* or *GLOGEN*. The ratio of proliferating cells rose to 27.73 ± 5.53% after functionalization with *GFOGER* (*P* < 0.01), 27.74 ± 7.02% with *GLOGEN* (*P* < 0.05) and 24.94 ± 3.57% with *VWFIII_Nle_* (*P* < 0.01). Combining *VWFIII_Nle_* with *GFOGER* (26.18 ± 6.58%, *P* < 0.05) or *GLOGEN* (25.04 ± 4.85%, *P* < 0.05) did not increase further proliferation rates. These findings demonstrate that HUVEC affinity and proliferation on collagen films can be enhanced by functionalization with THP ligands.

### 3D Collagen scaffold functionalization with THPs

**Figure 3:**
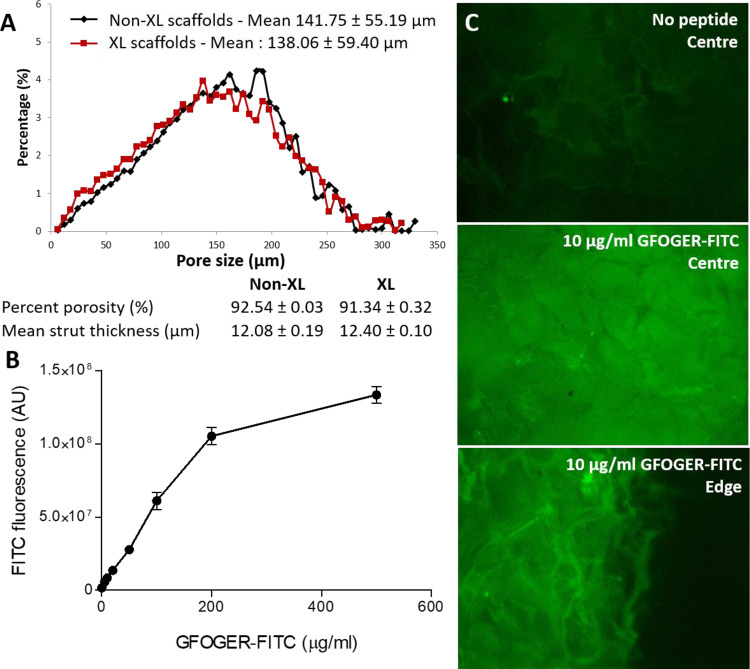
Scaffold characterization (**A**) Non-cross-linked (Non-XL) and EDC/NHS cross-linked (XL) collagen scaffold inner structure was analysed by X-ray microtomography. Pore sizes ranged from 0 to 280 µm and assumed a Gaussian distribution with an average size of 138–142 µm. Pore size and distribution were identical before and after EDC/NHS cross-linking. Non-cross-linked and cross-linked scaffolds had consistently high porosity of over 90% and mean strut thickness around 12 µm. (**B**) FITC fluorescence intensity in EDC/NHS cross-linked scaffolds incubated with *GFOGER–FITC* at concentrations ranging from 0 to 500 µg/ml. FITC fluorescence intensity linearly increased with *GFOGER–FITC* concentration up to 200 µg/ml before reaching saturation. (**C**) Representative fields of view of the *FITC–GFOGER* distribution in scaffolds loaded at 10 µg/ml. *GFOGER–FITC* was grafted homogeneously throughout scaffolds with no evident difference in fluorescence intensity between the edges and the centre.

3D collagen scaffolds were obtained by freeze–drying a collagen suspension. Pore dimensions and interconnectivity were determined by the size of interlocking ice crystals formed during the freezing process [29, 30]. The scaffold architecture was analysed by X-ray microcomputed tomography before and after EDC/NHS cross-linking. Non-cross-linked scaffolds were highly porous (92.54 ± 0.02% porosity), with a mean strut thickness of 12.08 ± 0.14 µm and a mean pore size of 141.75 ± 55.19 µm. Importantly, EDC/NHS cross-linking did not affect the porosity (92.18 ± 2.05%), the mean strut thickness (12.40 ± 0.07 µm) or the mean pore size (138.06 ± 59.40 µm). Furthermore, pore sizes assumed a Gaussian distribution that was unchanged by EDC/NHS treatment (Fig. 3A). 3D EDC/NHS cross-linked collagen scaffolds were loaded with a fluorescent derivative of *GFOGER* obtained by coupling FITC to the arginine side chain in each peptide strand (three FITC moieties per triple helix). *GFOGER–FITC* was introduced onto 2 mm thick cylindrical scaffolds at concentrations of 0, 5, 10, 20, 50, 100, 200 and 500 μg/ml. Scaffolds were gently compressed to ensure complete hydration and homogenous peptide distribution, exposed to UV light and extensively washed to remove non-covalently bound THPs. FITC fluorescence intensity increased linearly with the *GFOGER–FITC* concentration up to 200 μg/ml (linear regression fit *R*^2^ = 0.9702, Fig. 3B) before reaching a plateau. Finally, we verified that fluorescence intensity was identical at the centre and the edge of scaffolds, indicating that THPs were homogeneously distributed throughout these scaffolds following UV treatment (Fig. 3C). For the following experiments, scaffolds were initially functionalized with *GFOGER* at concentrations ranging from 5 to 200 µg/ml. However, no improvement in cell survival or invasion was detected with high peptide concentrations. In efforts to save materials, scaffolds were functionalized with 100 µl of THPs at 10 µg/ml, corresponding to 1 µg of THP per milligram of collagen (5 µg/ml for each THP; when only one active THP is present, 5 µg/ml of inactive GPP10 was added to achieve the same peptide loading density).

### HUVEC interactions with THP-functionalized 3D collagen scaffolds

**Figure 4:**
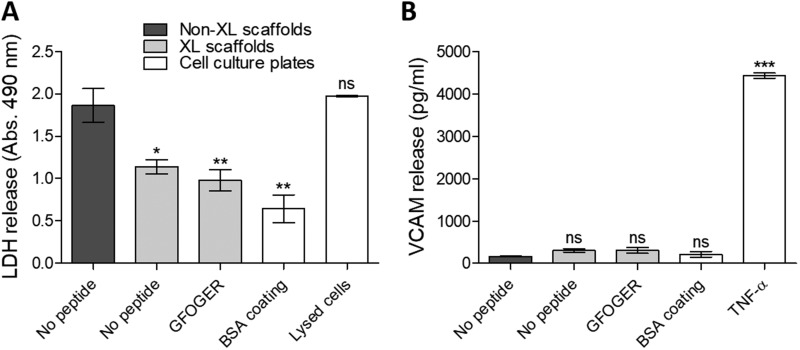
HUVEC phenotype 24 h after seeding on collagen scaffolds. Media from HUVEC cultures on non-cross-linked scaffolds (non-XL), EDC/NHS cross-linked scaffolds (XL) with no peptide or functionalized with *GFOGER* or cell culture plates coated with BSA was sampled after 24 h. (**A**) LDH activity measured by absorbance at 490 nm. Significance for each condition compared with non-cross-linked scaffolds is shown. Cross-linking prevented scaffold shrinking and degradation in culture media, and reduced cell death after 24 h. Covalent linkage of THPs did not cause additional cytotoxicity. (**B**) VCAM-1 secretion. Significance for each condition compared with non-cross-linked scaffolds is shown. Scaffold cross-linking and THP functionalization did not result in a dramatic increase in VCAM-1 release, indicating that scaffold preparation does not trigger pro-inflammatory activation.

HUVECs were seeded on non-cross-linked scaffolds, EDC/NHS cross-linked scaffolds with no peptide, GFOGER-functionalized EDC/NHS cross-linked scaffolds or BSA-coated cell culture wells. We first observed a dramatic contraction of non-cross-linked scaffolds in cell culture media (Supplementary Material) and important LDH secretion after 24 h (*A*_490nm_ = 1.85 ± 0.20, Fig. 4A, proportional to LDH released in media following cell death, one-way ANOVA, *P* < 0.001) almost identical as with lysed HUVECs (*A*_490nm_ = 1.97 ± 0.01). By contrast, with EDC/NHS cross-linked, scaffolds were more stable and cell death was lower (*A*_490nm_ = 1.15 ± 0.08, *P* < 0.05). These findings confirm the necessity of cross-linking collagen scaffolds to obtain stable materials that can provide a support for cells. Covalent linkage of GFOGER to the scaffolds did not increase LDH release into the media (*A*_490nm_ = 0.98 ± 0.13, *P* < 0.01), indicating that the THP-functionalization method is not a source of cytotoxicity for HUVECs. In parallel, we inspected whether scaffold preparation caused HUVEC activation into an inflammatory state by probing the culture media for soluble VCAM-1 (Fig. 4B, one-way ANOVA, *P* < 0.001). VCAM-1 concentration in the media from HUVECs cultured on EDC/NHS cross-linked scaffolds, with or without THPs (between 166.3 and 305.3 pg/ml), was not significantly different as for cells cultured on non-cross-linked films or BSA-coated tissue culture wells, and one order of magnitude lower than for HUVECs activated with TNF-α (4435.0 ± 60.7 pg/ml, *P* < 0.001). This implies that EDC/NHS cross-linking and THP functionalization does not have a pro-inflammatory effect on HUVECs.

**Figure 5:**
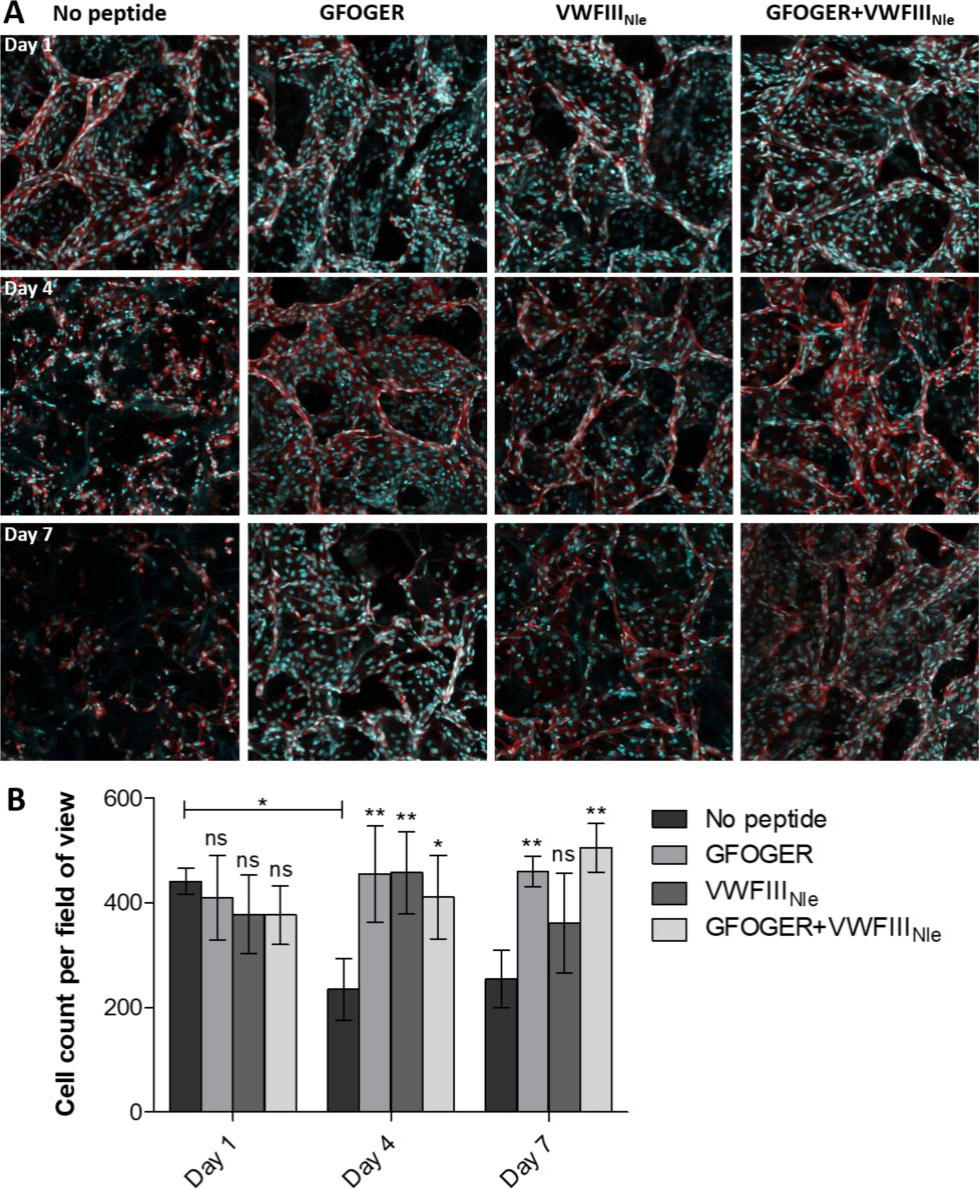
HUVEC seeding on top of collagen scaffolds. HUVECs were seeded onto the top surface of EDC/NHS cross-linked scaffolds without peptides or with *GFOGER*, *VWFIII_Nle_* or *GFOGER* + *VWFIII_Nle_*, cultured for 1, 4 or 7 days, fixed and stained with Hoechst 33342 (blue) and Rhodamine–Phalloidin (red). (**A**) Representative Z-stacks from plan views of the top 50 µm of scaffolds. HUVECs formed monolayers lining the inner pore surface after 1 day in all conditions, and after 4 and 7 days on scaffolds functionalized with THPs. (**B**) Cell number on top of scaffolds. Cell number dropped after 1 day without peptides but did not significantly vary in the presence of *GFOGER* and *GFOGER* + *VWFIII_Nle_*. Columns were compared using two-way ANOVA followed by Bonferroni’s multiple comparison tests. Significance for each condition compared with cross-linked scaffolds without peptide on the same day is shown.

Next, HUVECs were cultured over a period of 7 days on EDC/NHS cross-linked scaffolds without peptide or functionalized with *GFOGER*, *VWFIII_Nle_* and *GFOGER* in combination with *VWFIII_Nle_*. Cells on top of scaffolds were visualized using Hoechst 33342 and actin staining (Fig. 5A), and counted at day 1, 4 and 7 post-seeding (two-way ANOVA, *P* < 0.05, Fig. 5B). At day 1, there was no significant difference in cell count with or without THPs (440.92 ± 24.73 cells per field of view without peptides, 408.96 ± 80.71 with *GFOGER*, 377.75 ± 74.62 with *VWFIII_Nle_* and 376.29 ± 56.20 with *GFOGER* + *VWFIII_Nle_*). After 4 days, cell number significantly decreased at the cell-seeded surface on scaffolds without peptides (234.04 ± 59.50 cells per field of view) but was maintained in the presence of THPs (455.04 ± 91.66 cells per field of view, *P* < 0.01 for *GFOGER*, 457.00 ± 78.03, *P* < 0.01 for *VWFIII_Nle_* and 410.54 ± 79.91, *P* < 0.05 for *GFOGER* + *VWFIII_Nle_*). After 7 days, the cell density at the cell-seeded surface was preserved with *GFOGER* (459.42 ± 28.58, *P* < 0.01) and *GFOGER* in combination with *VWFIII_Nle_* (504.67 ± 46.31, *P* < 0.01), and significantly higher than in the absence of THPs (253.79 ± 54.83). *VWFIII_Nle_* gave inconsistent results between repeats and did not systematically guarantee a conserved cell population after 7 days (361.08 ± 95.10, not significant). Overall, cells were adherent after 24 h, forming monolayers covering the scaffold pore surfaces, but gradually became disorganized at day 4 and 7 with fewer cell–cell interactions in the absence of THPs. On functionalized scaffolds, however, the HUVEC monolayers persisted throughout the course of the experiment, populating the entire visible pore surface.

**Figure 6:**
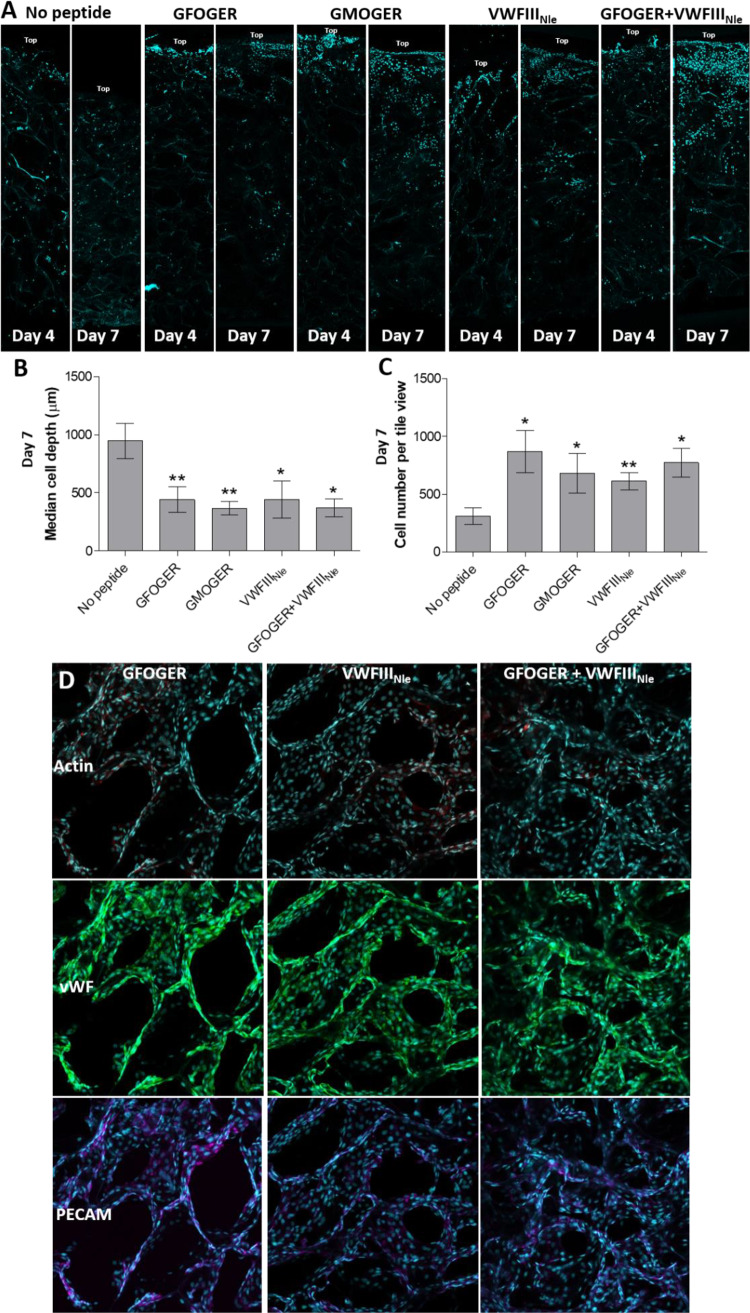
HUVEC Distribution in collagen scaffolds. HUVECs were seeded onto the top surface of EDC/NHS cross-linked scaffolds without peptides or with *GFOGER*, *GMOGER*, *VWFIII_Nle_* or *GFOGER* + *VWFIII_Nle_*, cultured for 4 or 7 days, fixed and stained with Hoechst 33342 (blue). (**A**) Representative tile views of scaffold cross-sections after 4 or 7 days of culture. Without peptides, HUVECs migrated into the scaffolds away from the seeding site. With THPs, HUVECs densely populated the seeding site at the top of scaffolds, with little migration and instead proliferated into adjacent pores. (**B**) Median cell depth after 7 days. Significance for each condition compared with cross-linked scaffolds without peptide is shown. Cell distribution was homogeneous on scaffolds without THPs, whereas most cells were located within the top half of the THP-functionalized scaffolds. (**C**) Cell number in cross-sections. Significance for each condition compared with cross-linked scaffolds without peptide is shown. Cell number was maintained over 7 days in THP-functionalized scaffolds and was significantly higher than scaffolds without peptides. (**D**) PECAM-1 (purple) and VWF (green) staining in HUVEC cultures on EDC/NHS cross-linked scaffolds functionalized with *GFOGER*, *VWFIII_Nle_* or *GFOGER* + *VWFIII_Nle_*. Representative Z-stacks from plan views of the top 30 µm of scaffolds are shown. PECAM-1 and VWF were detected in all cells, indicating that HUVECs cultured for 7 days on cross-linked scaffolds maintained an endothelial phenotype.

We then sought to determine whether the decrease in cell number in the absence of THPs was due to cell death or cell migrating away from the seeding site. Four or seven days post-seeding, scaffolds were cut longitudinally into two semi-circular pieces and cells were visualized along cross-sections (Fig. 6A). Cells generally penetrated inside the pores and slowly migrated throughout scaffolds without THPs. The median cell depth after 7 days (947.9 ± 150.8 µm, one-way ANOVA, *P* < 0.001, Fig. 6B) corresponded to half of the scaffold thickness, indicating a homogeneous distribution throughout the entire scaffold depth. However, few cells were present after 7 days (309 ± 71.08 cells per tile view, one-way ANOVA, *P* < 0.05, Fig. 6C). In contrast, cross-sections of THP-functionalized scaffolds showed poor HUVEC migration but higher cell number, suggesting enhanced cell survival (871.1 ± 182.4 (*P* < 0.05), 612.5 ± 74.66 (*P* < 0.01) and 772.9 ± 125.5 (*P* < 0.05) cells per tile view for *GFOGER*, *VWFIII_Nle_* and *GFOGER* + *VWFIII_Nle_*, respectively). HUVECs tightly associated with monolayers at a high cell density at the seeding site, that did not migrate but instead gradually proliferated into adjacent pores (441.6 ± 110.9 µm (*P* < 0.01), 443.8 ± 160.9 µm (*P* < 0.05) and 371.5 ± 75.4 µm (*P* < 0.05) median cell depth for *GFOGER*, *VWFIII_Nle_* and *GFOGER* + *VWFIII_Nle_*, respectively). We postulated that cell retention at the upper scaffold surface may be due to the high affinity between HUVECs and *GFOGER*, thereby minimizing migration. To explore this hypothesis, we functionalized scaffolds with *GMOGER*, a ligand with lower affinity for collagen-binding integrins. However, this resulted in equally limited cell migration (368 ± 59.9 µm median cell depth, *P* < 0.01) and similar cell count (680.0 ± 173.7 cells per tile view, *P* < 0.05). Finally, we verified that HUVECs cultured on scaffolds for 7 days did not lose their endothelial cell phenotype. PECAM-1 and VWF detected by immunofluorescence were consistently located in HUVECs regardless of the type of THP (Fig. 6D). This shows that HUVECs did not de-differentiate, but instead sustained a specific endothelial cell phenotype. Additionally, protein-level expression of PECAM and VWF suggests strong intercellular communication and functionality in HUVECs maintained on scaffolds for 7 days.

## Discussion

Amongst the wide range of biomaterials developed for hosting live cells, collagen-based matrices have been extensively used for tissue engineering and repair. Porous collagen scaffolds must have appropriate physical properties to maintain their structural integrity, prevent pre-mature degradation and mimic the native tissue as closely as possible. EDC/NHS cross-linking is commonly used to achieve adequate stiffness, but modifies collagen binding sites and results in weak cellular recognition [13, 31]. As a consequence, EDC/NHS cross-linked collagen is biologically inert to many cell types, including HT1080, Rugli [19], HEK293, Cos-7 and platelets [20]. Cellular response to biomaterials is directed by factors that include cell–receptor ligation, mechanical stimulation [32], growth factors [33] or the conditions of co-culture [34]. Here, we propose to use specific active sequences of native collagen that recognise collagen-binding proteins to improve cell–scaffold interactions and promote HUVEC culture and integration. These are especially important for endothelial cells which contact the basal lamina, composed mainly of collagen IV (along with collagen I, laminin, heparin, perlecan and fibronectin). Endothelial cells are central for vascularization and thus represent a pivotal cell lineage for tissue engineering. We thus aimed to promote endothelial cell survival, growth and function by controlling their interplay with collagen, by augmenting the scaffold with binding sites naturally occurring in the collagen sequence. HUVECs are the most widespread cell type used in endothelial research and have been extensively utilized to model vasculature and angiogenesis *in vitro*. They are readily available, cheap and are pooled from several donors when commercially supplied, limiting batch-to-batch variability. Consequently, we chose HUVECs as endothelial cell models in this study.

Ligands for collagen-binding integrins have been identified by our group over the past two decades [35]. These were included in THPs targeting preferentially α2β1 and α11β1 (*GFOGER*); preferentially α1β1 and α10β1 (*GLOGEN*) or all four integrins with moderate affinity (*GMOGER*). In addition, the sequence GPRGQOGVNleGFO in *VWFIII_Nle_* has been shown to bind DDR 1 and 2, SPARC and VWF. THPs containing these active sequences were end-stapled to stabilize the triple-helical conformation that is essential for biological activity, and coupled to diazirine for efficient covalent linkage to collagen upon UV treatment. Such THPs were previously employed to restore the cellular response of model cell lines that associate with collagen specifically via α1β1 and α2β1 [19], and DDR2 [20] on 2D collagen substrates. In this work, we applied this technique to HUVECs and transferred our methodology to 3D scaffolds. However, the cellular response to 3D scaffolds is complex, making mechanistic assessment of a single variable, for example, cell–receptor engagement, difficult. Therefore, preliminary experiments were carried out on 2D collagen films.

In our hands, non-cross-linked films shrank and dissolved in cell culture media. Adherent cells may pull, bend and rearrange the collagen surface [14], resulting in poor HUVEC spreading and low proliferation rate. Although HUVECs were adherent on EDC/NHS cross-linked films, they did not undergo full cellular spreading in the absence of THPs. Addition of *GFOGER* or *GLOGEN* significantly increased the mean cell surface area and was accompanied by visible actin polymerization and filopodia/lamellipodia projection. Integrin-binding THPs thus significantly improved the functional association of HUVECs with cross-linked collagen substrates. This led to enhanced cell proliferation after 24 h, rising from 11.9 to 25–28% with the addition of peptides, a value close to the proliferation rate observed in cell culture plates. HUVEC proliferation was thus improved by grafting THP ligands to the collagen substrate, without stimulating over-proliferation that could be problematic for applications in regenerative medicine. Interestingly, no significant variation was observed between different combinations of THPs. *VWFIII_Nle_* had the same impact as integrin-binding THPs, although the mechanisms involved remain unclear due to the ability of *VWFIII_Nle_* to bind several different proteins. Combining *VWFIII_Nle_* with *GFOGER* or *GLOGEN* did not alter the cell proliferative response, implying that *VWFIII_Nle_* and *GFOGER*/*GLOGEN* triggered overlapping signalling cues.

As THP functionalization showed promising results on 2D collagen materials, we transposed this methodology to porous 3D scaffolds. EDC/NHS cross-linked scaffolds with specified mechanical and structural properties have previously been fabricated in our laboratories [14, 38]. Adjusting the freeze–drying conditions creates 130–260 µm large and interconnected pores that are optimal for the diffusion, proliferation and self-organization of endothelial cells [36, 37]. Importantly, these architectural features, as well as pore size distribution and strut thickness, were not affected by EDC/NHS cross-linking. Such porous cross-linked collagen scaffolds have been populated with human fibroblasts [30] and KIM-2 mammary epithelial cells [38] without THP modification. Here, we have produced THP-functionalized 3D scaffolds that can host viable endothelial cells to provide biologically active biomaterials.

Initially, FITC conjugated to *GFOGER* was used to visualize the THP distribution in 3D scaffolds after UV-mediated derivatization. Bound *GFOGER–FITC* increased linearly with the coating concentration up to 200 μg/ml, before reaching a plateau, indicating saturation. FITC intensity was identical at the edges and at the centre of scaffolds showing that *GFOGER–FITC* was grafted uniformly throughout the scaffold mass. Using this protocol, scaffolds were functionalized with *GFOGER*, *VWFIII_Nle_*, *GFOGER* in combination with *VWFIII_Nle_* or *GMOGER*. Since *GLOGEN* and *GFOGER* showed identical HUVEC responses in 2D settings, only *GFOGER* was chosen for 3D experiments. To permit comparison between the 3D and 2D assays, the same THP concentration per mass of collagen was used. In *GFOGER*-functionalized scaffolds, we verified that cross-linking and subsequent UV treatment was not detrimental to cell viability. By contrast, non-cross-linked collagen scaffold degradation in overnight culture caused rapid cell death. Together with results obtained on non-cross-linked films, this confirmed the need to EDC/NHS cross-link collagen biomaterials. Scaffold preparation did not induce secretion of VCAM-1, which mediates the attachment of circulating blood leukocytes, potentiates an inflammatory reaction *in vivo* and initiates, in infection, the early stages of sepsis. Therefore, it is important that our method for THP-functionalization of EDC/NHS cross-linked scaffolds upon UV exposure did not result in cytotoxic or pro-inflammatory residual effects, indicating compatibility with applications in regenerative medicine.

When cultured on scaffolds for a week, HUVECs initially adhered to EDC/NHS cross-linked scaffolds and formed a monolayer covering the pore surface after 24 h, regardless of THP functionalization. Without peptides, HUVECs displayed gradual disorganization with less visible cell–cell contacts, accompanied by a decrease in cell number. Cross-sectional analysis showed that HUVECs in scaffolds without THPs were distributed homogeneously, migrating away from the seeding site. This confirms that our 3D collagen scaffolds possess sufficiently large and interconnected pores to allow endothelial cell migration. Conversely, the homogeneous cell layer across the scaffold’s pores at the seeding surface was retained on *GFOGER*- and *GMOGER*-decorated scaffolds for over a week in culture. *VWFIII_Nle_* led to variable outcomes between experimental repeats, while *GFOGER* in combination with *VWFIII_Nle_* yielded only a slightly higher cell number than *GFOGER* alone. This shows that integrin-binding THPs were required for maintaining endothelial cell layers over 7 days. As a result, slower cell migration and high cell density at the seeding site with progressive proliferation to populate adjacent pores were observed.

We theorize that the THPs, and in particular integrin-binding peptides, maintained cell survival through high-affinity interaction with HUVEC, hindering cell migration and preserving a densely populated layer at the site of seeding. *VWFIII_Nle_* also prevented migration, despite targeting proteins that are not considered to be adhesion receptors. As with films, we can only speculate on the mechanisms of action responsible for these results due to the promiscuity of *VWFIII_Nle_* for several collagen-binding proteins. Secreted VWF may bridge between HUVECs and scaffolds through binding of its A3 domain to the GPRGQOGVNleGFO sequence and its A1 domain to the GPIb complex or its RGD motifs in the C-domain to integrin αVβ3 on HUVECs [39]. Alternatively, *VWFIII_Nle_* may capture SPARC present in the media, preventing its interactions with HUVECs and thus diminishing its inhibitory effect on cell adhesion, spreading and proliferation [40]. *VWFIII_Nle_* may also bind to DDR2 present on the HUVEC surface and subsequently up-regulate integrin binding [17].

Finally, a frequent complication with long-term HUVEC culture is their de-differentiation into fibroblasts. We therefore examined the phenotype of HUVECs cultured for 7 days on THP-functionalized scaffolds by probing for PECAM-1 and VWF, both commonly used endothelial cell markers. VWF and PECAM-1 were present in all cells with no discernible difference between *GFOGER* or *VWFIII_Nle_* functionalization. VWF protein expression suggests that HUVECs remained functional 7 days after seeding. Moreover, PECAM-1 was present in intercellular HUVEC junctions on the THP-functionalized scaffolds, implying that they form a confluent endothelial layer with close intercellular communication [41]. Therefore, HUVECs preserved an endothelial cell lineage after one week in culture on THP-functionalized collagen scaffolds, encouraging us to believe that these novel biomaterials can be used in vascular tissue engineering.

## Conclusion

Robust protocols for the fabrication of porous collagen scaffolds with controlled architecture and mechanical properties have previously been established. Separately, we had covalently linked THPs to EDC/NHS cross-linked collagen films to enhance cell interactivity. This work combines the two technologies by transposing our THP functionalization methodology to 3D collagen scaffolds. Active biomaterials decorated with THPs targeting collagen-binding integrins, VWF, DDRs and SPARC were designed to host HUVECs. THPs improved HUVEC spreading and proliferation on collagen films and proved to be essential for cell survival and cellular cross-talk for 7 days in scaffolds. Importantly, HUVECs were strongly adherent to THP-functionalized cross-linked collagen, formed monolayers across pore surfaces, progressively populated pores surrounding the seeding site with high cell density and preserved an endothelial phenotype. These findings establish THP-functionalized 3D collagen materials as promising cell substrates to produce vascularized engineered tissues. Such biomaterials have great potential for tissue engineering applications, either as *in vitro* platforms to study endothelial cell behaviour or as implants intended to be grafted at the site of injury *in vivo* to initiate tissue regeneration and revascularization.

## Supplementary Material

rbaa025_Supplementary_Data
