# Supplementary material for: Collagen scaffolds functionalized with triple-helical peptides support 3D HUVEC culture
Source: Regen Biomater. 2020 Aug 18;7(5):471–82. doi: 10.1093/rb/rbaa025 (PMC7597804; doi:10.1093/rb/rbaa025)
Supplement: rbaa025_Supplementary_Data [file rbaa025_supplementary_data.docx]

**Supporting Information.**

Collagen scaffold shrinkage in cell culture media.


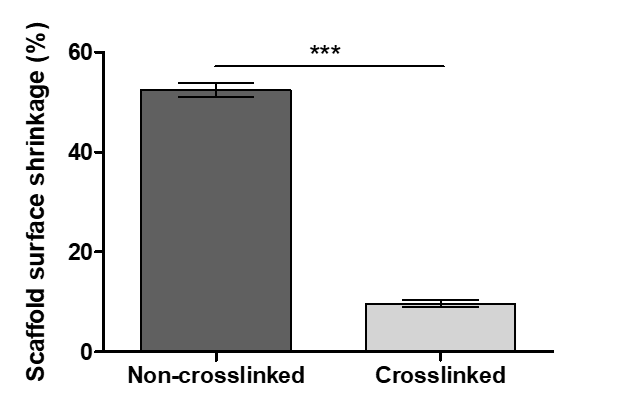


Non-crosslinked and EDC/NHS crosslinked cylindrical scaffolds were left in cell culture media (DMEM) for one week. The top of disc-shaped surfaces of scaffolds were measured at day 0 and day 7. The percentage of top surface shrinkage in three experimental repeats was calculated. Non-crosslinked scaffolds showed dramatically more contraction (52.49 ± 1.37 % shrinkage) than crosslinked scaffolds (9.86 ± 0.67 % shrinkage, one-way ANOVA, p < 0.001).
